# What causes the experience of discrimination in non-regular workers?

**DOI:** 10.1186/s40557-017-0192-x

**Published:** 2017-08-15

**Authors:** Seong-Hoon Kang, Jin-Ho Song, Tae Hwan Koh, Do Myung Paek, Jong-Tae Park, HoSun Chun

**Affiliations:** 1 Department of Occupational and Environmental Medicine, Korea University Ansan Hospital, Ansan, South Korea; 2 Department of Occupational and Environmental Medicine, School of Public Health, Seoul National University, Seoul, South Korea

**Keywords:** Discrimination, Non-regular workers, Occupational stress

## Abstract

**Background:**

Discrimination based on type of employment against non-regular workers is still a social issue. However, there are few studies on job factors that affect the discrimination experience in each type of employment or the association between discrimination and health impact indicators. This study examined occupational health characteristics according to discrimination experience and relating factors that affect discrimination experience.

**Methods:**

This study used the 4th Korean Working Conditions Survey (2014) provided by the Korea Occupational Safety and Health Agency. Among the 50,000 workers, 7731 non-regular wage workers were selected as study population. To examine differences in discrimination experience, we used a t-test on occupational risk factors, occupational stress, occupational characteristics, health impact indicators. To identify the factors that affected discrimination experience, we performed binomial logistic regression analysis.

**Results:**

The discrimination experience rate was significantly higher in male, aged less than 40 years old, above high school graduate than middle school graduate, higher wage level, shorter employment period and larger company’s scale. As factors related to discrimination experience, they experienced discrimination more as occupational stress was higher and when they were temporary or daily workers rather than permanent workers, work patterns were not consistent, and the support of boss was low. It showed that physical, musculoskeletal, and mental occupational risk scores and subjective job instability were higher and work environment satisfaction was lower in discrimination experienced group.

**Conclusions:**

The present study showed that the demographic and occupational factors were complexly related to discrimination experience in non-regular workers. The experience of discrimination had increased when occupational stress was higher, they were temporary or daily workers rather than permanent workers, work patterns were not consistent, and their boss’ support was low. Improving various relating factors, (e.g. occupational stresses, employment status and occupational characteristics), this would ultimately expect to improve non-regular workers’ discrimination.

## Background

According to the “Supplementary Survey for the Economically Active Population Survey” conducted by Statistics Korea, the proportion of non-regular workers remained at around 55–65% from August 2001 to March 2007, began decreasing from August 2007, and reached 43.6% in March 2016 [1]. The proportion is still high compared to the OECD average of 11.1% and the average of 14.4% in European countries [2]. In addition, 96.1% of non-regular workers are either temporary workers or workers who have temporary job elsewhere; hence job stability in Korea can be considered very weak in comparison to that in other countries [1].

In general, non-regular workers can be classified according to type of employment [3]. Classification based on type of employment defines non-regular labor as labor in which the objectives in the contract concluded with the employer differ from that of conventional regular work. Based on this definition, hourly, temporary, daily, and dispatched labor is classified as non-regular labor [3–5]. In fact, there are various types of employment within the non-regular labor category, and because of these, sub-groups can be significantly dissimilar even within the same category [6]. Furthermore, a large gap in working conditions is evident between sub-groups in the non-regular worker group depending on the industry and type of employment [7]. According to the type of employment in non-regular workers, the additional study is requested for the discrimination in the viewpoint of occupational health level.

Discrimination refers to unfair treatment experienced by an individual or group based on their affiliation or status [8]. Discrimination based on type of employment is a good example. According to the recent studies, non-regular workers mostly have poorer working conditions in terms of wages, additional wages, employee benefits, and social security as well as job stability than do regular workers. Furthermore, it is not easy to transition from non-regular employment to regular employment, which tends to be increasingly firmly entrenched [9]. Even in the public sector, a large gap is evident in working conditions between non-regular and regular workers, such as in wages and employee benefits, and it was found that they are frequently given unreasonable work orders [10]. In addition, non-regular workers were reported to have a lower level of health than regular workers across various health indicators such as chronic disease, acute disease, social physiological health, and self-rated health [11]. Sub-contract workers are more likely to experience occupational accidents than regular workers, and some studies have pointed out non-regular employment as a major reason for the occurrence of occupational accidents [12, 13].

In this regard, the law prohibits discrimination based on type of employment. The Act on the Protection, Etc. of Fixed-Term and Part-Time Employees and the Act on the Protection, Etc. of Dispatched Workers state that the employer should not discriminate against fixed-term, part-time (hourly), and dispatched workers in terms of wages and other working conditions without reasonable justification compared to regular workers performing the same or similar work in the business or workplace. These Acts prevent discrimination against non-regular workers based on type of employment and protect their appropriate working conditions. However, discrimination is quite common nonetheless.

Most previous studies on non-regular workers focused on identifying gaps in working conditions such as wages, social security, job security, differences in job satisfaction, and inequality in health by comparing to regular workers [9, 10, 14, 15]. Although there were some studies to investigate on the gaps among non-regular worker, they have focused only on socioeconomic status such as wages or employee benefits, but neglected on health and safety issues. In addition, there are few studies on job factors that affect the experience of discrimination in each type of employment or the association between discrimination and health impact indicators [16, 17]. As such, we intended to examine occupational health characteristics according to discrimination experience and relating factors that affect discrimination experience in the non-regular workers. This study ultimately aimed to determine measures to improve non-regular workers’ discrimination experience and contribute to establishing occupational health policies.

## Methods

### Subjects

This study used the 4th Korean Working Conditions Survey (2014) provided by the Korea Occupational Safety and Health Agency. For the survey subjects, this study extracted sample households from apartment and general enumeration districts in the “Population and Housing Census” as the survey population, and selected those who met the employee criteria in the extracted households. The survey method was a one-on-one interview by a professional interviewer conducted through door-to-door visits. The questionnaire comprised questions related to the work environment, occupational characteristics, working hours, organizational communication, social psychological factors, health impact indicators, job satisfaction, and demographic characteristics.

Among the 50,000 workers in the 4th Korean Working Conditions Survey, 7731 workers who identified themselves as non-regular wage workers when responding to KQ78-d questions were selected as the study population. We excluded regular wage workers (22707), self-ownership without employee (13815), self-ownership with employee (3240), unpaid family workers (2409). When missing value for each questions existed, they were excluded in our analysis.

### Methods

#### General characteristics

This study used sex, age, education, and income as sociodemographic factors, and the scale and characteristics of a company, working hours per week, and the employment status as occupational characteristics.

The age of respondents was classified as 16 to 29 years, 30 to 39 years, 40 to 49 years, and 50 years and older. The education level was categorized into middle school graduation or lower, high school graduation, and college graduation or higher. Subjects’ average income was divided into less than 0.75 million won, 0.75 to 1.49 million won, and 1.5 million won or more.

Next, the scale of the company subjects worked for was categorized into less than 5 employees, 5 to 9 employees, and 10 employees or more. The company’s characteristics were categorized as private sector, public sector, private-public partnership organization, and non-profit organization. Subjects’ average working hours per week was categorized into less than 30 h, 30 to 44 h, and 45 h or more. Employment status was divided into permanent, temporary, and daily workers.

#### Definition of variables

This study used one question among the work environment questions or developed and used a proxy indicator by combining similar topic questions.

Discrimination experience used J of Question 65, which asked about the experience of discrimination in each type of employment.

Subjective job instability employed Question 75, overall work environment satisfaction Question 76, presenteeism Question 74, threat to health Question 66, and overall health status Question 68. A higher score means a higher level of subjective job instability, work environment satisfaction, and threat to health, but a poorer level of overall health status.

In this study, physical occupational risk factors, musculoskeletal occupational risk factors, and mental occupational risk factors were scored on a seven-point scale based on the level of exposure in each question, and the sum of the points was used for the analysis. A higher score means a higher level of exposure to risk factors.

Work patterns were to see the whether the form of employment was consistent or not. As for it, this study assigned 0 points to “agree” and 1 point to “disagree” for A, B, C, and D in Question 37 (about whether work patterns were consistent), while 1 point was assigned to “agree” and 0 points to “disagree” for E and F, which concerned stand-by work or shift work. This study analyzed the sum of these points. A higher score means work patterns are not consistent.

For questions related to occupational stress factors, this study referred to the 4^th^European Working Conditions Survey and the Korean Occupational Stress Scale (KOSS) for its categorization. Each question related to job demand, job autonomy, interpersonal conflict, job instability, and lack of reward was scored on a five-point scale and the sum of points was used for the analysis in this study. A higher score means a higher level of job demand, interpersonal conflict, and job instability, and a lower level of job autonomy and reward. The total score of occupational stress was calculated by summation of values of five parameters. Depending on whether support of boss was supportive, this study used “yes” as 0 points and “no” as 1 point. A higher score means support of boss was not supportive.

Proxy indicators used for each topic are described in Table 1. As mentioned earlier, the range of scores for each proxy indicator was from one to seven for physical occupational risk factors, musculoskeletal occupational risk factors, mental occupational risk factors, zero to one for work patterns, support of boss, and one to five for occupational stress, respectively. We also presented a cronbach’s alpha for each proxy indicators. Except mental occupational risk factors, internal consistency reliability was adequate by each proxy indicator.

**Table 1:** Proxy indicators used for each topic

Proxy indicator	Questionnaire	Cronbach’s alpha
Physical occupational risk factors	Q23 A, B, C, D, E, F, G, H, I	0.812
Musculoskeletal occupational risk factors	Q24 A, B, C, D, E	0.648
Mental occupational risk factors	Q24 F, G	0.449
Work patterns	Q37 A, B, C, D, E, F	0.686
Support of boss	Q58 A, B, C, D	0.811
Occupational stress- job demand	Q45 A, B	0.882
Occupational stress- interpersonal conflict	Q51 A, B	0.811
Occupational stress- job autonomy	Q51 E, F, O	0.632
Occupational stress- job instability	Q77 A, F	0.524
Occupational stress- lack of reward	Q77 B	

### Statistical method

SPSS version 20.0 was used as the statistics program. (SPSS, Chicago, IL, USA) To analyze subjects’ general and occupational characteristics, this study resorted to descriptive statistics. To examine differences in discrimination experience, this study used a t-test on work environment satisfaction, physical, musculoskeletal, and mental occupational risk factors, occupational stress, work patterns, support of boss, threat to health, overall health status, presenteeism, and the number of absenteeism. This study adjusted for parameters that are known to influence discrimination experience and statistically significant in uni-variable logistic linear regression analysis. (e.g. sex, age, education level, wage, average working hours per week, and the company’s scale and characteristics) And it performed binomial logistic regression analysis to identify the factors that affected discrimination experience. This study verified statistical significance based on α = 0.05.

## Results

### Characteristics and discrimination experience of the subjects

Among the subjects, male workers accounted for 41.8% and female workers 58.2%. The average age was 48.33 years and the largest age group was those aged 50 years or older, who accounted for 49.9% of the subjects. For education level, the largest group was those who graduated from high school (47.9%), followed by 31.1% for those with middle school graduation or lower, and 16.6% for those with college graduation or higher. The average income was 1.235 million won, and workers with 0.75 to 1.49 million won in income accounted for the highest percentage at 36.1%. Regarding the company’s scale, companies with 10 or more employees accounted for the highest percentage (39.3%), followed by those with less than 5 employees and those with 5 to 9 employees. The average working hours per week was 37.42, and less than 30 working hours accounted for the highest percentage (35.4%). The average working period was 64.0 months. For employment status, temporary workers accounted for the highest share at 54.6%, followed by daily workers (26%) and permanent workers (19.3%) (Table 2).

**Table 2:** Characteristics and discrimination experience of the subjects

Characteristics	Discrimination experience (%)	Non discrimination experience (%)	Total (%)	Chi-squared test
Sex				<0.00
Male	281 (8.8)	2929 (91.2)	3210 (41.8)	
Female	246 (5.5)	4216 (94.5)	4462 (58.2)	
Age (years)				0.009
16–29	119 (8.5)	1284 (91.5)	1403 (18.3)	
30–39	63 (7.3)	795 (92.7)	858 (11.2)	
40–49	100 (6.3)	1482 (93.7)	1582 (20.6)	
≥ 50	245 (6.4)	3584 (93.6)	3829 (49.9)	
Education level				0.04
Middle school	135 (5.7)	2227 (94.3)	2362 (31.1)	
High school	266 (7.3)	3372 (92.7)	3638 (47.9)	
College	116 (7.3)	1476 (92.7)	1245 (16.6)	
Average income(Won)				0.01
< 750,000	132 (5.8)	2160 (94.2)	2292 (30.6)	
750,000–1,499,999	186 (6.9)	2522 (93.1)	2708 (36.1)	
≥ 1,500,000	191(7.6)	2308 (92.4)	2499 (33.3)	
Company’s scale				0.005
< 5	151 (5.4)	2648 (94.6)	2799 (38.5)	
5–9	111 (6.8)	1510 (93.2)	1621 (22.3)	
≥ 10	214 (7.5)	2643 (92.5)	2857(39.3)	
Company’s characteristics				0.001
Private sector	456 (7.4)	5736(92.6)	6192(81.7)	
Public sector	51 (4.7)	1034 (95.3)	1085 (14.3)	
Non-profit organization	14 (4.7)	285 (95.3)	299(3.9)	
Working hours per week (hours)				0.107
< 30	185 (6.9)	2488 (93.1)	2673 (35.4)	
30–44	156 (6.1)	2393 (93.9)	2549 (33.8)	
≥ 45	178 (7.7)	2148 (92.3)	2326 (30.8)	
Duration of employment (months)				0.001
< 12	204 (8.5)	2195 (91.5)	2399 (31.3)	
12–47	136 (6.1)	2090 (93.9)	2226 (29.0)	
≥ 48	187 (6.1)	2860 (93.9)	3047 (39.7)	
Employment status				<0.00
Permanent workers	76 (5.2)	1398 (94.8)	1474 (19.3)	
Temporary workers	263 (6.3)	3903 (93.7)	4166 (54.6)	
Daily workers	186 (9.4)	1798 (90.6)	1984 (26.0)	

The discrimination experience rate was significantly higher in male, aged less than 40 years old, above high school graduate than middle school graduate, higher wage level, shorter employment period and larger company’s scale. There was no statistically significant difference in discrimination experience according to the average working hours.

### Working conditions and health impact indicators based on discrimination experience

The subjective job instability score of the group who experienced discrimination was 1.97, which was significantly higher than that of 1.82 of the ones who have not. Likewise, work environment satisfaction score was 2.32 in the group who experienced discrimination, which was significantly lower than that of 2.63 of the ones who have not.

Regarding occupational risk factors, the group who experienced discrimination scored 18.86, 17.41, and 5.19 in physical, musculoskeletal, and mental occupational risk factors respectively, all significantly higher than the 16.23, 15.63, and 4.93 of the ones who have not.

Regarding occupational stress, the group who experienced discrimination scored 5.98, 10.84, 5.91, 5.56, and 3.25 for job demand, job autonomy, interpersonal conflict, job instability, and lack of reward respectively, all significantly higher than the 4.93, 10.37, 5.61, 5.12, and 3.07 of the ones who have not. The sum of the occupational stress variables was significantly higher for the discrimination group.

For occupational characteristics, the group who experienced discrimination scored 1.35 and 2.36 for work patterns and support of boss respectively. These points were significantly higher than those of the ones who have not, demonstrating that work patterns were not consistent and the support of boss was lower for the discrimination group.

Regarding health impact indicators, the group who experienced discrimination scored 0.31, and 0.29 in the level of threat to health and presenteeism respectively, significantly higher than the ones who have not. Overall health status and the number of absenteeism scored 2.48 and 0.89 in the group who experienced discrimination, and although it was higher than that of 2.38 and 0.72 of the ones who have not, it was not statistically significant (Table 3).

**Table 3:** Working conditions and health impact indicators based on discrimination experience

	Discrimination experience	Non discrimination experience	T-test
Effect			
Subjective job instability	1.97 ± 0.72	1.82 ± 0.70	<0.00
Work environment satisfaction	2.32 ± 0.69	2.63 ± 0.62	<0.00
Occupational risk factors			
Physical	18.86 ± 9.23	16.23 ± 7.63	<0.00
Musculoskeletal	17.41 ± 5.56	15.63 ± 5.40	<0.00
Mental	5.19 ± 2.80	4.93 ± 2.76	0.04
Occupational stress			
Job demand	5.98 ± 2.55	4.93 ± 2.36	<0.00
Job autonomy	10.84 ± 2.25	10.37 ± 2.28	<0.00
Interpersonal conflict	5.91 ± 1.60	5.61 ± 1.70	<0.00
Job instability	5.56 ± 1.43	5.12 ± 1.51	<0.00
Lack of reward	3.25 ± 0.93	3.07 ± 0.91	<0.00
Total	31.77 ± 4.82	29.23 ± 4.80	<0.00
Occupational characteristics			
Work patterns	1.35 ± 1.64	1.05 ± 1.46	<0.00
Support of boss	2.36 ± 1.82	1.88 ± 1.84	<0.00
Health impact indicators			
Threat to health	0.31 ± 0.46	0.16 ± 0.37	<0.00
Overall health	2.48 ± 0.76	2.38 ± 0.73	0.06
Presenteeism	0.29 ± 0.45	0.21 ± 0.41	0.001
The number of Absenteeism	0.89 ± 13.65	0.72 ± 7.12	0.643

### Factors associated with discrimination experience

When adjusting for sex, age, education level, wage, average working hours per week, and the company’s scale and characteristics, the OR (CI) (odds ratio (95% confidence interval)) of the group with a high level of occupational stress as 3.25 (2.28–4.63) and that of the group with a middle level of occupational stress as 2.31 (1.62–3.28). The groups with high and middle levels of occupational stress were 3.25 and 2.31 times more likely to experience discrimination than the group with a low level of occupational stress. Temporary and daily workers’ OR (CI) was 1.45 (1.05–2.05) and 1.83 (1.21–2.76) respectively. Temporary and daily workers were 1.45 and 1.83 times more likely to experience discrimination than permanent workers. When work patterns were not consistent, the OR (CI) was 1.35 (1.05–1.73). Support of boss was not supportive, the OR (CI) was 1.33 (1.03–1.72) (Table 4).

**Table 4:** Factors associated with discrimination experience

	Adjusted OR (95% CI)^a^
Occupational stress	
High level	3.25(2.28–4.63)
Middle level	2.31(1.62–3.28)
Low level	1.00
Employment status	
Permanent workers	1.00
Temporary workers	1.45(1.05–2.05)
Daily workers	1.83(1.21–2.76)
Work patterns	1.35(1.05–1.73)
Support of boss	1.33(1.03–1.72)

^a^Adjusted for sex, age, education level, wage, working hours per week, and the company’s scale and characteristics

## Discussion

In this study, the discrimination experience rate was significantly higher in male, aged less than 40 years old, above high school graduate than middle school graduate, higher wage level, shorter employment period and larger company’s scale. This demonstrates that various factors such as sex, age, education level, wage, years of employment, and the company’s scale are involved in combination when the worker perceives discrimination. Since there are few existing studies on the factors or mechanisms affecting non-regular workers’ discrimination experience, it is difficult to directly compare or interpret this study’s results. However, preceding researches findings that male and high wage workers were more likely to be exposed to occupational risk factors in non-regular workers [1], and only because on non-regular workers whose income averages at about 1.235 million won in our research, they are more likely demanding high intensity of work and harsh working environment for more wage. So it could be interpreted as such that poor working conditions marked by increased exposure to occupational risk factors have led to having felt discriminated against. This interpretation would be consistent with results from another study that working conditions have an effect on discrimination experience [18]. In addition, women tend to deny their discrimination experience in the workplace more than men and underreport their discrimination experience; therefore, men seem to experience more discrimination [19, 20]. When the education level is higher, the reporting of discrimination experience tends to increase [21]. As the degree to which workers accept social disadvantages arising from their employment conditions could vary, it is possible that they are affected by personal traits in addition to objective working conditions. The results that workers experienced more discrimination when relatively younger in age, have above high school graduate than middle school graduate, and the company’s scale was larger may be due to differences in such personal social standards by which any feeling, including that of discrimination is gauzed, but follow-up research is required to further explore the correlations.

For working conditions according to discrimination experience, the group who experienced discrimination had a higher level of physical, musculoskeletal, and mental occupational risk factors. It seems that poor working conditions affected their discrimination experience, which is consistent with the findings of a previous study that discrimination experience was affected by working conditions [18]. For health impact according to discrimination experience, it was found that the group who experienced discrimination had a higher threat to health and presenteeism. These results are comparable to those of other studies, in feeling of having discriminated could act as both physical and mental stressor that raise blood pressure, increase the occurrence of cardiovascular diseases, cause mental illness such as depression and increase unhealthy behaviors, which in turn would ultimately deteriorate overall health status [22–26].

To identify the factors that affected discrimination experience, this study adjusted for sex, age, education level, wage, average working hours per week, and the company’s scale and characteristics by using binomial logistic regression analysis.

The group who experienced discrimination scored higher for occupational stress, and the one with a high level of occupational stress experienced discrimination 2.31 to 3.5 times more than the one with a low level. In previous studies, non-regular workers had more occupational stress factors than regular workers, a higher level of job demand and job instability, and a lower level of job autonomy, and their compensation system was not appropriate [12, 27, 28], in short such studies have shown that differences in working conditions between the two groups led to differences in the level of occupational stress. It could be considered that discrimination experience could have also influenced occupational stress. However, it is more proper to understand that occupational stresses, such as high level of job demand, interpersonal conflict, job instability and low level of job autonomy, rewards, have increased the incidences of discrimination experiences. But follow-up research is required to further explore the correlations between occupational stress and discrimination experience. As this study found that the percentage of discrimination experience increased among sub-groups in the non-regular group when the level of occupational stress increased, occupational stress must be managed by focusing on those workers who experienced discrimination.

Regarding employment status, temporary and daily workers experienced discrimination 1.45 and 1.83 times more than permanent workers. These results seem to stem from the fact that temporary and daily workers’ job stability is inferior to that of permanent workers, even though they all are, in essence, non-regular workers, which can be substantiated by the results of another study that have shown the level of exposure to risk factors to be different even among non-regular workers, depending on the type of employment: fixed-term, non-fixed term, hourly, temporary agency, and indirect [1]. Differences in job stability and risk factors affected discrimination experience among non-regular workers. In Working Conditions Survey, among non-regular workers, employment status was further classified to permanent (employed longer for a year), temporary (employed longer than a month, but shorter than a year), daily (employed shorter than a month). And among non-regular workers, differences in discrimination experience existed, depending on the status of employment. As such, in order to make a policy for reducing discrimination experiences more efficiently, it is necessary to subclassify non-regular workers.

When work patterns were not consistent, it was about 1.4 times more likely that discrimination would be experienced. It is reckoned that inconsistent working hours and shift work can cause insomnia and interfere with family and social life [29], or a high level of occupational stress from shift work could have an effect [30]. Authors of this study assert that inconsistent work patterns decrease the quality of life while increasing occupational stress and affects discrimination experience.

When support of boss was low, it was about 1.3 times more likely that discrimination would be experienced. Previous studies indicated that the support of boss decreased role conflict and physical stress while boosting confidence [31, 32], and was a protection factor that decreased the experience of a sense of depression [33, 34]. Therefore, the support of boss is considered a factor that decreases discrimination experience, because the boss has more tools and means within the workplace to handle the situation and access to institutional support for workers who experience discrimination [35].

It was found that the group who experienced discrimination had a high level of subjective job instability and a low level of work environment satisfaction. This decreases an individual worker’s productivity, and job commitment and responsibility, which consequently reduces organizational productivity as a whole [36, 37]. Preceding study also suggested that subjective perception of job stability was correlated to job commitment [4]. Thus, it seems sound that aiming to reduce discrimination experience, which could in turn affect subjective job stability to increases job commitment might help to improve collective productivity of organization. Only, this may be influenced by discrimination experiences, but it may affect the experience of discrimination. Follow-up research is required to further explore the correlations.

This study examined occupational health characteristics according to discrimination experience and relating factors that affect discrimination experience. It is worth noting that this study is meaningful in that it presented basic data for policies to reduce discrimination experience of non-regular workers. However, the original purpose of data of the Working Conditions Survey used in this study was to investigate working conditions and identify various forms of risk factors; thus, there were limitations in terms of analyzing factors related to discrimination experience in more depth based on such data. And the information bias might be presented because workers who responded more having discrimination tend to respond having worse outcomes. But to control possible bias, Working Conditions Survey tried to minimize systemic or information bias by implementing random sampling and one on one interview. And it was comprised of ninety questions, of which only one (65th) pertained to the discrimination, the possibility seems to be low for the discriminated workers to respond having worse outcomes. Nevertheless, as this study was cross-sectional, it was limited in that it was difficult to identify a causal relationship and to say that there is no information bias. Also, owing to Working Conditions Survey used in this study was subjective questionnaire, the possibility of exaggerated or unintentional incorrect response cannot be excluded. As such, possible of misclassification for exposures and outcomes cannot be excluded. Therefore, a future study would need to be conducted to supplement this limitation.

## Conclusion

Using data of the 4th Korean Working Conditions Survey, this study examined occupational health characteristics according to discrimination experience and relating factors that affect discrimination experience. The demographic and occupational factors were complexly related to discrimination experience in non-regular workers. The experience of discrimination had increased when occupational stress was higher, they were temporary or daily workers rather than permanent workers, work patterns were not consistent, and their boss’ support was low. Improving various relating factors, (e.g. occupational stresses, employment status and occupational characteristics), this would ultimately expect to improve non-regular workers’ discrimination.

## Abbreviations

CI: Confidence interval
OR: Odds ratio
